# A Novel Conserved Isoform of the Ubiquitin Ligase UFD2a/UBE4B Is Expressed Exclusively in Mature Striated Muscle Cells

**DOI:** 10.1371/journal.pone.0028861

**Published:** 2011-12-09

**Authors:** Andrew L. Mammen, James A. Mahoney, Amanda St. Germain, Nisha Badders, J. Paul Taylor, Antony Rosen, Sarah Spinette

**Affiliations:** 1 Johns Hopkins University School of Medicine, Department of Medicine, Division of Rheumatology, Baltimore, Maryland, United States of America; 2 Johns Hopkins University School of Medicine, Department of Neurology, Baltimore, Maryland, United States of America; 3 Department of Developmental Neurobiology, St. Jude's Children's Research Hospital, Memphis, Tennessee, United States of America; 4 Department of Biology, Rhode Island College, Providence, Rhode Island, United States of America; Instituto Butantan, Brazil

## Abstract

Yeast Ufd2p was the first identified E4 multiubiquitin chain assembly factor. Its vertebrate homologues later referred to as UFD2a, UBE4B or E4B were also shown to have E3 ubiquitin ligase activity. UFD2a function in the brain has been well established *in vivo*, and *in vitro* studies have shown that its activity is essential for proper condensation and segregation of chromosomes during mitosis. Here we show that 2 alternative splice forms of UFD2a, UFD2a-7 and -7/7a, are expressed sequentially during myoblast differentiation of C2C12 cell cultures and during cardiotoxin-induced regeneration of skeletal muscle in mice. UFD2a-7 contains an alternate exon 7, and UFD2a-7/7a, the larger of the 2 isoforms, contains an additional novel exon 7a. Analysis of protein or mRNA expression in mice and zebrafish revealed that a similar pattern of isoform switching occurs during developmental myogenesis of cardiac and skeletal muscle. In vertebrates (humans, rodents, zebrafish), UFD2a-7/7a is expressed only in mature striated muscle. This unique tissue specificity is further validated by the conserved presence of 2 muscle-specific splicing regulatory motifs located in the 3′ introns of exons 7 and 7a. UFD2a interacts with VCP/p97, an AAA-type ATPase implicated in processes whose functions appear to be regulated, in part, through their interaction with one or more of 15 previously identified cofactors. UFD2a-7/7a did not interact with VCP/p97 in yeast 2-hybrid experiments, which may allow the ATPase to bind cofactors that facilitate its muscle-specific functions. We conclude that the regulated expression of these UFD2a isoforms most likely imparts divergent functions that are important for myogenisis.

## Introduction

The way in which cells achieve their differentiation program is intimately tied to changes in their proteome occurring via several mechanisms including, post-translational modification, *de novo* transcription, ubiquitin dependant proteasomal degradation, and specific regulation of tissue-specific splicing factors resulting in alternatively spliced transcripts. Several recent genome wide screens have estimated that over 90% of all genes are alternatively spliced and that more than 50% of all alternative splicing events differ by tissue type, with muscle displaying the 3^rd^ highest number of alternatively spliced transcripts [1], [2], [3]. Between 60 and 95 distinct splicing transitions have been detected during cardiac development and skeletal muscle differentiation more than half of which are conserved between mammalian and avian species [4], [5]. Troponins, titin, MEF-2, and MBNL proteins represent specific examples of developmentally regulated splicing events which affect protein function, localization and/ or binding specificity in these tissues [6], [7], [8], [9].

Proteasome-dependant protein turnover is also critical for the dramatic alterations in the proteome that occur during myogenesis. The temporal regulation of muscle-specific transcriptional events is regulated by the dependant degradation of myogenic transcription factors such as MyoD and Myf5 and their regulatory cofactors [10], [11], [12], [13]. In addition, multiple structural components of the sarcomere are substrates of the ubiquitin proteasome system (UPS) [14], [15]. During development, ubiquitin-dependant degradation facilitates myosin heavy chain (MHC) isoform switching [16] and the is crucial for turnover of myosin binding chaperones that affect overall sarcomere assembly [17], [18], [19], [20].

The UPS is a multi-enzyme, ATP-dependent process which generally requires three enzymes: an E1 ubiquitin activating enzyme, an E2 ubiquitin conjugating enzyme, and an E3 ubiquitin protein ligase. These enzymes catalyze the covalent attachment of the ubiquitin polypeptide to a target protein, followed by the attachment of further ubiquitin peptides onto already attached ubiquitin. The majority of the target protein specificity is mediated at the level of the E3 ligase, of which, well over one hundred examples have been described from three main gene families [21], [22]. In some cases, multi-ubiquitination requires the additional activity of an E4 ligase, which binds to proteins with just a few ubiquitin molecules and catalyses multiubiquitin-chain assembly in collaboration with E3 ligases. Polyubiquitinated proteins are then targeted for proteolytic destruction by the proteasome.

UFD2a (also referred to as E4B/Ube4b) is an E3/4 ubiquitin ligase that we and others have recently characterized in vertebrates [23], [24], [25]. The carboxy terminus of UFD2a contains a U-box which is related to the more common RING domain [26] and was shown to contain the active site for UFD2a ubiquitin ligase activity as well as the binding site for the two E2 enzymes with which it associates, UbcH5c and Ubc4 [24], [25], [27], [28], [29], [30]. We later determined that a novel amino terminal domain, the MPAC (Mitotically Phosphorylated, Apoptotically Cleaved), not present in lower eukaryote UFD2a orthologs, was also required for full E3 ligase activity [28].

In human cells, RNAi knockdown of UFD2a led to aberrant chromosomal condensation and segregation, mitotic arrest and apoptosis, demonstrating that UFD2a was essential for proper progression through mitosis. Examination of UFD2a function *in vivo* has mainly focused on its role in the central nervous system (CNS). Specifically, UFD2a is implicated in spinocerebellar ataxia type-3 (SCA) [27] and UFD2a ubiquitination activity is required for normal CNS development [31]. Overexpression of UFD2a in transgenic mice resulted in accumulation of ubiquitin containing aggregates in hypothalamic neurons, which led to significant metabolic abnormalities and obesity [32]. The only evidence for UFD2a function in other tissues *in vivo,* was shown in mice homozygous for the U-box deletion which died *in utero* of massive cardiomyocyte apoptosis [31]. However, the mechanism of UFD2a involvement in muscle tissue development or function has not been characterized.

Here we identify two cardiac and skeletal muscle-specific isoforms of UFD2a, UFD2a-7 and UFD2a-7/7a. We show that these isoforms arise from alternative splicing of the UFD2a pre-mRNA and include one and two additional exons, respectively, compared to the ubiquitously expressed isoform of UFD2a. The expression and tissue specificity of these alternative transcripts, as well as the presence of several muscle specific intronic regulatory motifs in intron 7 appear to be conserved among several vertebrate species including human, mouse, rat and zebrafish. Interestingly, while UFD2a-7/7a is expressed in mature and regenerating muscle, UFD2a-7 is expressed only transiently during muscle regeneration *in vivo*. We find that this pattern of isoform switching also occurs during embryonic development in mice. Finally, the mature muscle specific isoform, UFD2a-7/7a, fails to bind to the multi-functional AAA type ATPase, VCP/p97, an interaction that has been well documented for UFD2a across species.

## Results

### A novel form of UFD2a is expressed in skeletal muscle and cardiac muscle

**Figure 1:**
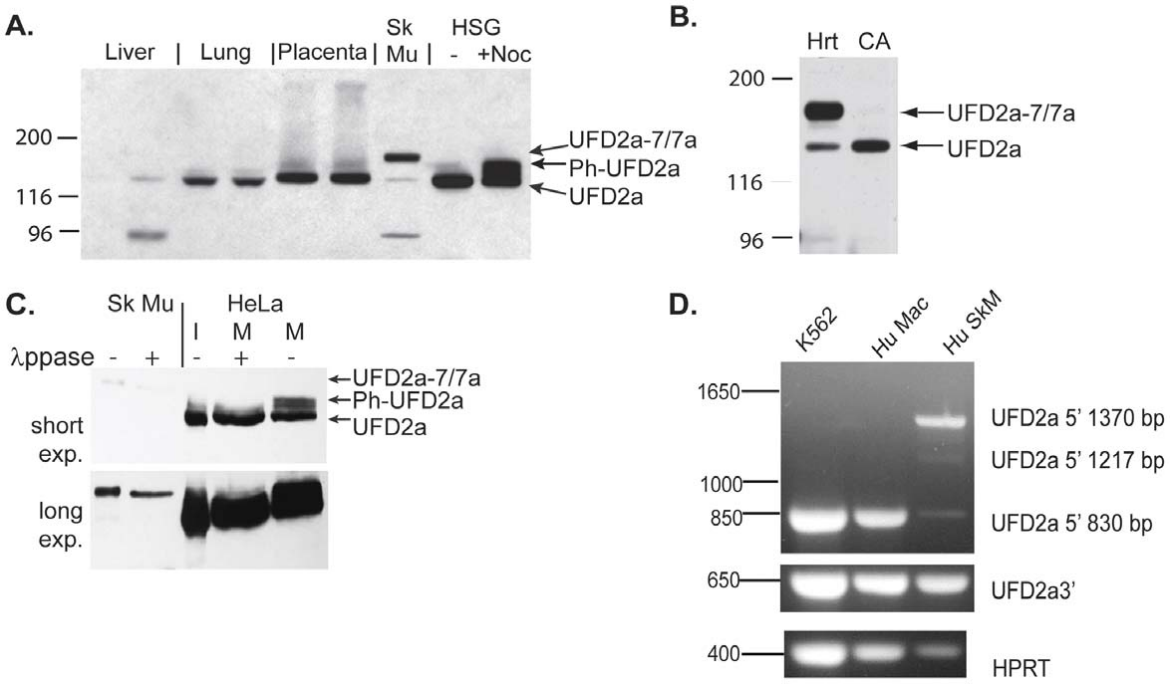
A larger isoform of UFD2a is specifically expressed in striated muscle. A). Protein lysates were prepared from normal human tissues and human salivary gland cells (HSG) that were untreated (-) or arrested in mitosis (+Noc) and subjected to SDS PAGE. After transfer to nitrocellulose, samples were analyzed by immunoblotting using a rabbit polyclonal antibody recognizing UFD2a. A 97-kDa band visible in many muscle lysates and some other tissues represented cross-reactivity to phosphorylase b (not shown). B). UFD2a-7/7a expression in cardiac muscle (Hrt) but not coronary artery (CA) from an explanted human heart (3 patient samples were analyzed; 1 is shown here). C). Human skeletal muscle (Sk Mu) lysates and lysates made from human HeLa cells arrested in mitosis (M) with nocodazole were treated with λprotein phosphatase (λppase). Interphase (I) and untreated mitotic (M) HeLa cells are shown for comparison; phos refers to the phosphorylated form of UFD2a. Top and bottom panels represent different exposures of the same Western blot to account for the difference in the UFD2a levels in total protein from cell cultures compared to human tissue. D).Total RNA from human skeletal muscle (Hu SkM), K562 human erythroleukemia cells, and primary human monocyte-derived macrophages (Hu Mac) were used as templates for cDNA reactions. RT-PCR analyses were performed using primers recognizing a portion from the 5′-end of UFD2a (upper panel), the 3′-end of UFD2a (middle), or from the housekeeping gene *HPRT* (lower). The 5′ UFD2a primers amplified the expected 830-bp fragment from other tissues and the novel 1217-bp and 1370-bp fragments in muscle.

Previous studies by our group and others have shown that UFD2a is widely expressed in multiple cell types and tissues in vertebrates [24], [25]. However, Western blot analysis revealed that UFD2a expressed in skeletal muscle migrated significantly slower on SDS-PAGE than in other tissues, suggesting a muscle tissue–specific alternate isoform (Fig. 1A). Western blot analysis of other muscle tissues revealed that the larger isoform is also expressed in human cardiac tissue (Fig. 1B). Coronary arteries, which are an abundant source of smooth muscle, were analyzed from the same donors, but only the ubiquitous form of UFD2a was expressed in that tissue. Small intestine, another prominent source of smooth muscle, also expressed only the ubiquitous form (not shown), suggesting that the larger isoform of UFD2a is specifically expressed in striated muscle. We have previously shown that UFD2a is highly phosphorylated in mitotic cells, which increases its molecular size [23], [28]. However, the muscle-specific isoform did not comigrate with phosphorylated UFD2a from cells arrested in G2/M with nocodazole (upper band of doublet seen in lane 9 Fig. 1A), and unlike the mitotic phosphorylated forms of UFD2a [23], [28], the muscle-specific form was insensitive to treatment with λ protein phosphatase (Fig 1C, long exposure).

### The muscle-specific form of UFD2a includes 2 isoforms with alternatively spliced exons

RT-PCR of muscle and nonmuscle cDNA revealed the presence of skeletal muscle-specific expanded Ufd2a cDNA sequence. Multiple primer pairs were used to amplify different portions of the UFD2a cDNA. A primer pair specific for sequences near the 5′-end of the UFD2a gene amplified an expected 830-bp fragment of cDNA derived from human primary macrophages and transformed erythroblast cells. However, a dominant 1370-bp fragment, and one 1217-bp intermediate, in addition to trace amounts of the expected 830-bp fragment were detected in human muscle cDNA (Fig. 1D and data not shown). RT-PCR using primers that recognize a sequence nearer the 3′-end of the UFD2a gene, as well as those recognizing the housekeeping gene HPRT, amplified the same-sized product from all sources examined.

**Figure 2:**
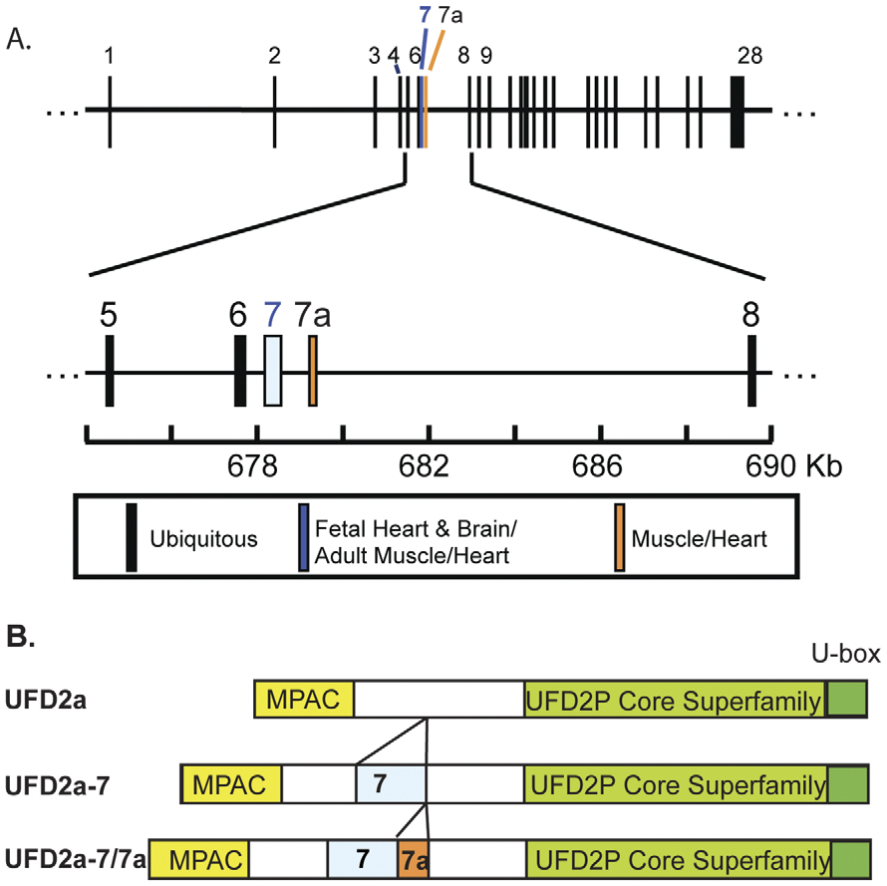
Positions of the muscle-specific exons. A). This map shows the locations of the 29 exons of the human *UFD2a* gene spread across approximately 200 kb near the telomere on the short arm of chromosome 1. Ubiquitous exons are shown in black. Exon 7, which has been found in fetal brain and striated muscle, is shown in blue. The striated muscle–specific exon 7a is show in red. B). Illustration of the UFD2a protein showing positions of the MPAC domain, catalytic U box, exons 7 and 7a, and the region conserved in lower eukaryotes (light green).

Muscle and nonmuscle RT-PCR products were analyzed by sequence analysis. Nonmuscle amplicons matched exactly the UFD2a RefSeq entry (Accession NM_006048) in the NCBI sequence database. The dominant 1370 bp muscle amplicons (largest band in Fig. 1D) contained the same sequence with an additional 540 bp of novel sequence. The novel sequence was inserted after amino acid 270 within the N-terminal region not present in lower eukaryotes such as yeast and drosophila Ufd2, just downstream of the MPAC regulatory region [28]. Alignment of this extended muscle-specific sequence with genomic sequences from human chromosome 1 (Accession NC_000001) demonstrated the presence of 2 additional exons in the muscle-specific isoform that were 387 bp and 153 bp in length (Fig. 2A); both exons maintained the open reading frame. The 387-bp sequence corresponding to exon 7 was originally found in a UFD2a cDNA clone derived from a human fetal brain cDNA library, entered into the NCBI database in 1998 (Accession AF043117), and later was referred to as transcript variant 1 (RefSeq Accession NM_001105562). However, when we cloned UFD2a from HeLa cell cDNA, exon 7 was not present (RefSeq Accession NM_006048) [24]. We will hereafter refer to the UFD2a isoform containing exon 7 spliced to exons 6 and 8 as UFD2a-7.

Interestingly, two recent genome-wide exon-junction and whole transcript microarrays, which examined the expression of alternative pre-mRNA splice forms across a total of 92 tissues and cell lines, both found that exon 7 was not present in UFD2a cDNA from human fetal brain tissue. In fact, consistent with the data presented here, exon 7 was exclusively expressed in adult human skeletal muscle, heart, tongue (therefore, predominantly in striated muscle cells) and to a lower extent peripheral blood leukocytes. Of the nine fetal tissues tested, only fetal heart expressed exon 7 (fetal skeletal muscle was not included) [1], [2].

Since the 153-bp exon has not yet been reported, we termed this novel exon, which immediately follows exon 7 in muscle transcripts, exon 7a. Exons 7 and 7a are flanked by canonical splice-donor and -acceptor sequences. As a result of the inclusion of exons 7 and 7a, the muscle-specific isoform of UFD2a, which we have termed UFD2a-7/7a (submitted to Genbank, Accession JF289274), contains 180 additional amino acids (Fig. 2B).

### Conservation and tissue specificity of UFD2a-7 and UFD2a-7/7a in vertebrates

**Figure 3:**
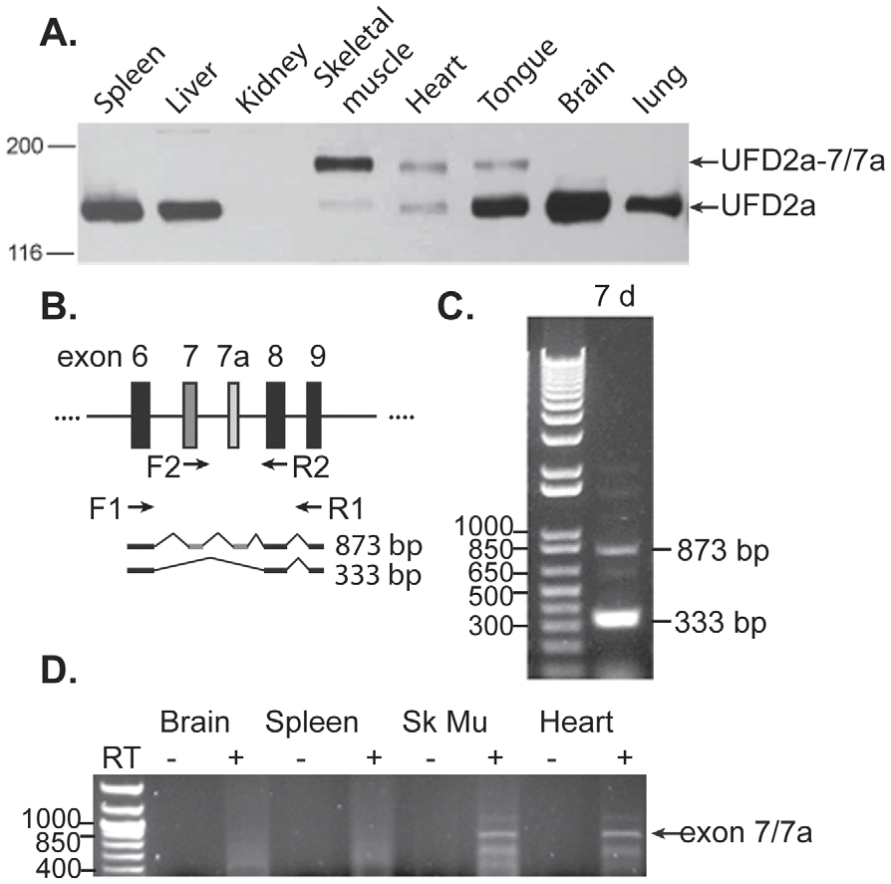
Expression of the muscle-specific alternative isoforms of *UFD2a* is conserved in mice and zebrafish. A). Among 8 murine tissues, UFD2a-7/7a is expressed only in striated muscle. Protein lysates prepared from adult murine tissues analyzed by immunoblotting using a rabbit polyclonal antibody recognizing UFD2a. B). Schematic diagram depicting the exons of zebrafish *ube4B* (UFD2a ortholog) and the location of various primer sets used for RT-PCR. C). RNA was extracted from whole 7 pfd zebrafish embryos and subjected to RT-PCR analysis using primers F2 and R2. The 333-bp band represents the fragment expected from the amplification of exons 6, 8, and 9, and the 873-bp fragment includes amplification of the suspected exons 7 and 7a. D). RNA extracted from 4 tissues from multiple adult zebrafish was analyzed by RT-PCR using primers F2 (specific for exon 7) and R2 (exon 8).

To examine the tissue specificity of UFD2a-7 and -7/7a, we prepared protein extracts from various murine tissues and analyzed their expression via Western blot. Among the 9 tissues, only the heart, tongue, and skeletal muscle contained the approximately 163-kD UFD2a-7/7a form (Fig. 3A). These tissues also appeared to contain a variable amount of ubiquitous UFD2a, though this result is likely due to the presence of nonmuscle cells (e.g., fibroblasts and erythrocytes) in the dissected tissues. As shown previously, UFD2a expression was almost absent in the kidney [25].

Embryonic striated muscle development is easily viewed in zebrafish, and morpholino oligonucleotides have been used extensively in this organism to examine the significance of alternative transcripts. Thus, we sought to determine whether UFD2a-7 and -7/7a are expressed in zebrafish. RT-PCR was performed on RNA extracted from whole zebrafish at 7 days postfertilization (dpf) by using primers F1 and R1, which flank the predicted locations of exons 7 and 7a (Fig 3B), according to *ube4b* (*UFD2a* ortholog) nucleotide entries NP_919343 and NM_194362. Most of the resulting cDNA product was 333 bp, representing the amplification of the ubiquitous form of *ube4b* contained within this primer set; this product did not contain exons 7 and 7a (Fig. 3C). However, there was also a faint, larger (873 bp) band present that presumably corresponded to the inclusion of exons 7 and 7a predicted via homology to mammalian *UFD2a* exons (Fig 3C).

The tissue specificity of the Ube4b isoform containing exons 7 and 7a was examined by RT-PCR using primer R2 (Fig 3B) and a primer specific for the newly identified exon 7. Among brain, spleen, skeletal muscle, heart (shown in Fig 3D), testes and liver (not shown) extracted from multiple adult fish, a product containing exon 7 and 7a was only obtained from skeletal muscle and heart cDNA, suggesting that the tissue specificity of these exons are conserved in zebrafish.

### The intron downstream of exons 7 and 7a contains two conserved motifs associated with exons upregulated in striated muscle

**Figure 4:**
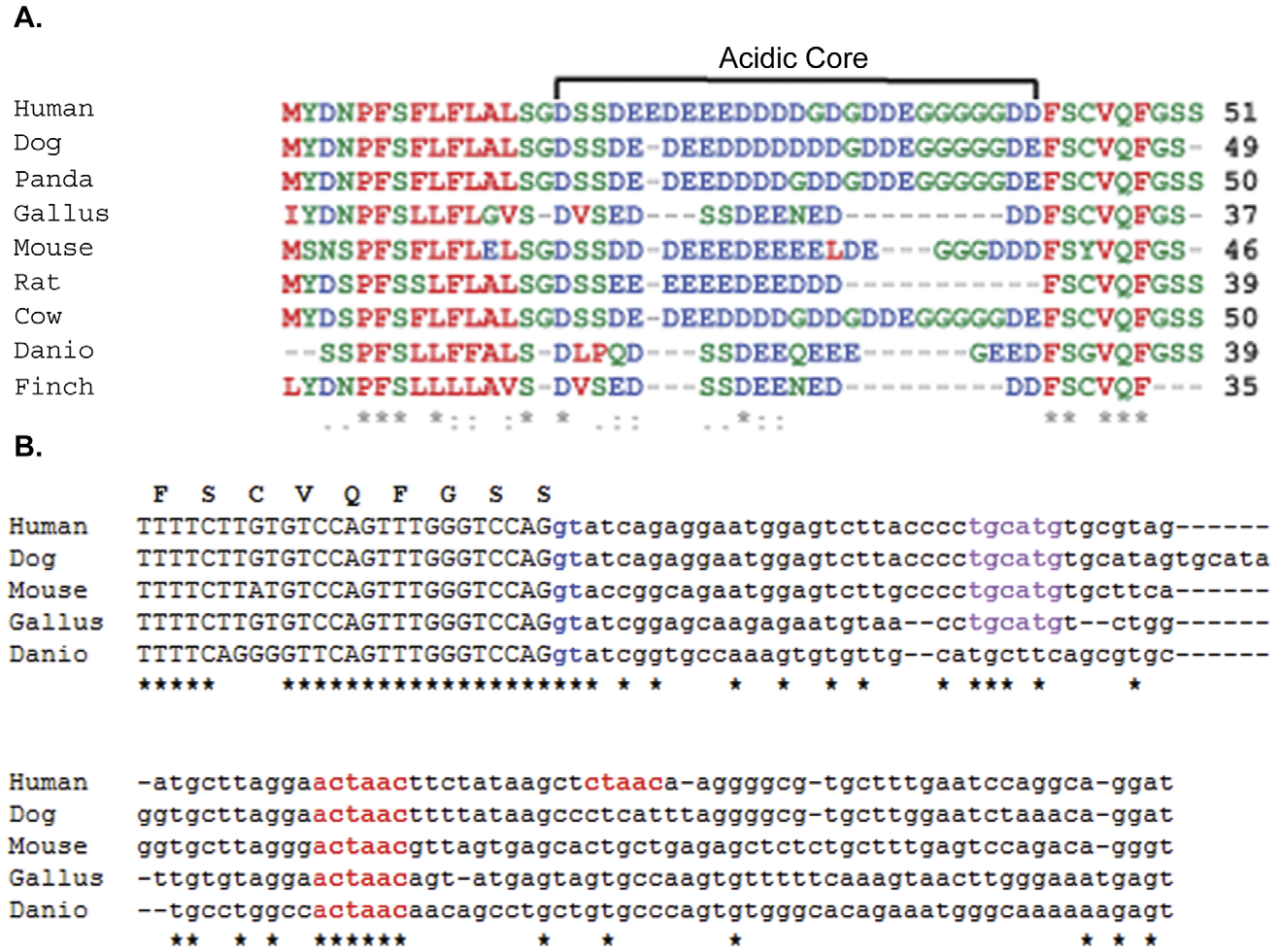
*UFD2a* exon-7a is highly conserved across vertebrates and contains conserved motifs associated with muscle-specific alternative splicing. A). Alignment of exon 7a amino acid sequence. Asterisks indicate complete conservation across all species, and colons indicate strong conservation. Negatively charged residues are shown in blue, and hydrophobic residues are shown in red. B). Alignment of the 3′-end of exon 7a plus the first 100 bp of the following intron. The splice donor site is shown in blue. The CTAAC putative muscle-specific alternative splicing signal is shown in red. The TGCATG Fox motif enriched in introns of striated muscle exons is shown in purple. Residues identical in all sequences are marked with an asterisk. The amino acid sequence of human exon 7a is shown above.

Multiple sequence alignments of exons 7 (not shown) and 7a (Fig. 4A) from 9 vertebrate species revealed a high degree of sequence identity. In exon 7, the main variability was seen in a slightly degenerative repeat region in mice that is not present in the other organisms examined. Variation within exon 7a existed mainly in the length of a highly acidic, low-complexity central core, primarily composed of aspartic acid, glutamic acid, serine, and glycine. Sequences flanking this central core were more highly conserved.

Recent studies taking advantage of microarrays and new computational techniques have begun to elucidate patterns in the expression of alternatively spliced genes. A study by Sugnet, et al [33] showed that many alternatively spliced exons that are preferentially expressed in muscle tissue exist upstream of a novel sequence motif, CTAAC, within the first 150 nucleotides of the downstream intron. A similar analysis of alternative splicing events occurring during myogenic differentiation identified ACTAAC as a regulatory sequence, which is conserved in introns flanking alternatively spliced exons in 7 mammalian species [4]. Examination of the intron downstream of UFD2a exon 7a revealed 2 exact matches to these motifs, nearly in tandem, approximately 50 bp downstream of the splice donor (Fig. 4B).

Another recent screen investigated the presence of regulatory motifs enriched in introns adjacent to alternatively spliced exons among tissue-specific, alternatively spliced genes in humans. In this study, the TGCATG (FOX-1) motif was highly enriched 10 to 80 nts downstream of skeletal muscle and heart specific exons with limited or no expression in other tissues (p<10^−16^) [1]. The introns downstream of both exon 7 (not shown) and 7a (Fig 4B) in the human UFD2a sequence contain a TGCATG FOX-1 motif at position+21 and 26 respectively. This sequence was not present in any other 3′-intronic regions, suggesting the specificity of this motif for directing the alternative splicing of these exons.

If muscle-specific alternative-splicing motifs are required to maintain accurate muscle-specific expression patterns, then there would be selective pressure to maintain those motifs across species. Thus, we used CLUSTALW [34], [35] to align across 5 vertebrate species the sequences downstream of exon 7a (Fig. 4B). Among the *UFD2a* genomic sequences analyzed, a copy of the ACTAAC motif was located about 50 bp downstream of the end of exon 7a; the TGCATG motif was found in 4 of the 5 species but was not present in zebrafish. In addition, examination of all of the 3′-flanking intronic regions (100 nts) within the *UFD2a* genomic sequence across the 5 species revealed that the ACTAAC motif occurred only 6 additional times, and the specific intron in which it was found was not conserved. This suggests that the presence of the ACTAAC motif in other introns was not under selective pressure and may be a result of random assortment. Also, the intronic sequences, with the exception of these 2 motifs, were poorly conserved among the species as expected.

### Expression of UFD2a-7 and UFD2a-7/7a are sequentially induced upon muscle differentiation

Mature muscle tissue is generally postmitotic, where little or no cell division is detected in mature myofibers. Replacement of damaged or senescent muscle fibers occurs by proliferation and differentiation of satellite cells, a tissue-resident muscle cell precursor population (for reviews: [36]–[37] and [38]). We previously showed that UFD2a plays a central role in mitotic progression [23]; thus, we investigated whether UFD2a, UFD2a-7, or UFD2a-7/7a is expressed in immature muscle cells and/or during muscle cell maturation.

**Figure 5:**
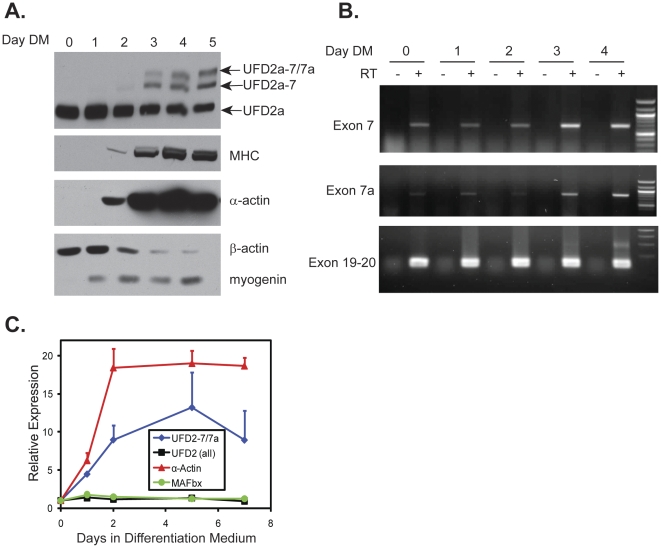
UFD2a-7 and UFD2a-7/7a mRNA and protein expression are upregulated during differentiation and regeneration. A). C2C12 cells were plated in growth medium overnight and then transferred into differentiation medium (DM). Cell cultures were harvested for analysis after the indicated times. Equal total protein was loaded on SDS-PAGE (see Figure S1 for poneau S staining) and analyzed for expression of the indicated proteins. The UFD2a panel was performed on a large-format 6% bis-acrylamide gel for greater separation of the 2 larger UFD2a isoforms and probed with the 15041 polyclonal antibody, which recognizes all isoforms. B). C2C12 cells were plated in growth medium overnight and then transferred into DM. Cell cultures were harvested for analysis after the indicated times. Total RNA was isolated using TRIzol and used as a template for RT-PCR. PCR was performed on the resulting cDNA (made with or without reverse transcriptase (RT)) using a 5′ primer complementary to exon 6 and a 3′ primer specific for either the exon 7/exon 8 junction (top panel) to detect UFD2a-7 transcripts or the exon 7a/exon 8 junction (middle panel) to detect UFD2a-7/7a transcripts. Primers specific to exon 19 and 20 were used in the bottom panel to show total *UFD2a* transcripts. C). Total RNA was isolated with TRIzol from duplicate samples of cells used in panel A and subjected to RT-PCR. Quantitative PCR was performed in duplicate using TaqMan primers that recognized the indicated transcripts. Transcript expression data, normalized to the expression of HPRT, are expressed relative to the expression level at time zero. Error bars indicate standard deviation.

Differentiation of the C2C12 murine myoblast cell line is a well-established model of maturation from myoblasts to myotubes [39], [40]. Only the ubiquitous isoform of UFD2a was expressed in the dividing myoblasts (Fig. 5A; days 0–1). Interestingly, upon differentiation, 2 larger (by apparent MW) isoforms were sequentially induced. Specifically, UFD2a-7 appeared at very low levels by Day 2 and increased by Day 3, while UFD2a-7/7a appeared at Day 3 and increased on subsequent days (note that equal total protein loading across lanes is shown in Fig S1). Induction of these larger isoforms occurred with timing similar to the appearance of mature muscle markers such as myosin heavy chain (MHC), muscle-specific α-sarcomeric actin, and creatine phosphokinase (Fig. 5A and data not shown). In addition, RT-PCR using a forward primer spanning the junction between exons 6 and 7 with a reverse primer spanning exons 7 and 8 showed that transcripts containing only exon 7 (*UFD2a-7*) were apparent at appreciable levels in undifferentiated cells (Days 0–1) and then greatly increased at days 3 and 4, when the cultures contained multinucleated myotubes (Fig. 5B; top panel). In contrast, use of a reverse primer specific for the junction between exons 7a and 8 in conjunction with the previous exon 6–7 junction forward primer showed that transcripts containing 7a are present at very low levels until Day 3 (Fig. 5B; middle panel). RT-PCR using primers specific for exons 19 and 20 revealed that the total amount of UFD2a transcripts (all three isoforms) appeared to be relatively equal at all time points (Fig. 5B, bottom panel).

In contrast with mature muscle tissue, differentiating C2C12 cells expressed the intermediate-sized isoform, UFD2a-7. When maximal differentiation into myotubes occurred (Day 5), only about 30% of the total UFD2a protein expression consisted of the upper 2 isoforms corresponding to UFD2a-7 and -7/7a (Fig. 5A top panel). However, the continued expression of myogenin (Fig. 5A), a bHLH family transcription factor important in myoblast development [41], provided evidence that this system does not lead to fully mature myofibers. Therefore the data show that UFD2a-7 expression precedes that of UFD2a-7/7a and suggests that it may occur transiently during differentiation, while the *exclusive* expression of UFD2a-7/7a is a late event in myogenesis, occurring at a stage that cannot be replicated in C2C12 cell cultures.

Quantitative RT-PCR showed that at the mRNA level, the *UFD2a-7/7a* isoform was detectable in the undifferentiated C2C12 mouse myoblasts but was induced approximately 10-fold upon differentiation into myotubes (Fig. 5C). Total *UFD2a* mRNA, representing isoforms with or without exons 7 and 7a, remained relatively constant. As expected, α-sarcomeric actin was also strongly induced during C2C12 myoblast differentiation, while the expression of muscle-specific E3 ligase atrogin-1/MAFbx mRNA was unchanged. The finding that *UFD2a-7/7a* message was detectable in undifferentiated C2C12 myoblasts implies that the expression of UFD2a-7/7a protein may also be subject to translational control, as previously shown for the muscle-specific transcription factor MyoD [11]. Alternatively, UFD2a-7/7a may be rapidly degraded in proliferating myoblasts, thereby leading to steady-state protein levels too low for detection by Western blotting.

### UFD2a isoform expression is regulated in a mouse model of muscle regeneration

**Figure 6:**
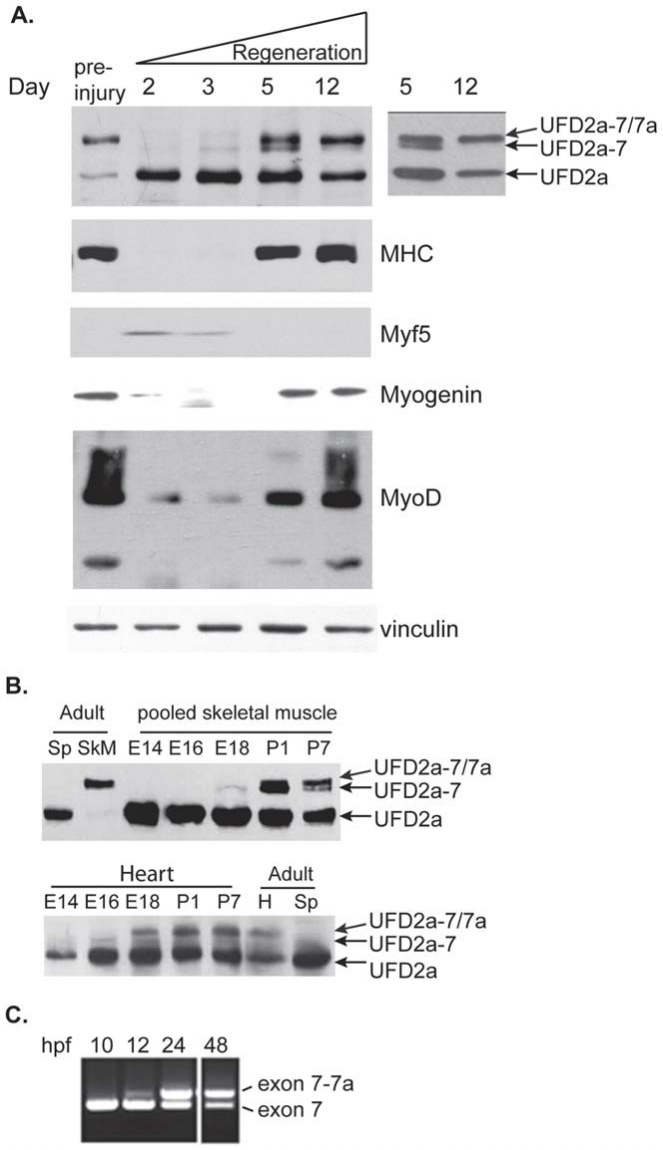
Expression of UFD2a alternative-splice forms occurs sequentially during regeneration and developmental myogenesis. A). Mice received intramuscular injections of cardiotoxin (CTX) in the right tibialis anterior muscle. After the indicated number of days of recovery, the treated and untreated contralateral muscles were harvested and analyzed by immunoblotting for UFD2a, the mature muscle marker myosin heavy chain (MHC), the proliferating myoblast marker Myf5, myogenin, MyoD, and vinculin as a loading control. The inset at right shows the exposure from a low-bis gel in which the UFD2a-7 isoform of intermediate size is more clearly separated from UFD2a-7/7a at Day 5 and more clearly shows its absence at Day 12 (arrows point to the two isoforms). B). Skeletal muscle (upper panels; pooled gastrocnemius, soleus, and quadriceps muscles) or hearts (lower panels) from E14, E16, and E18 embryos and P1 and P7 pups were dissected from individual embryos or pups at each time point and analyzed by Western blotting for UFD2a (note a second set of mice are shown in Fig S2). Adult mouse heart (H), skeletal muscle (SkM), and spleen (Sp) were simultaneously analyzed for comparison. C). RNA was extracted from whole zebrafish embryos at 10, 12, 24, and 48 hpf, and nested PCR was performed using primers F3 and R3 on the 873- and 756-bp fragments produced from the initial RT-PCR.

To probe the regulation of UFD2a isoforms in response to muscle damage *in vivo*, we used the cardiotoxin (CTX) model of muscle degeneration/regeneration in mice [42]. CTX, a cobra venom peptide, is a pore-forming toxin that potently targets muscle cells. We injected the toxin along the entire length of the tibialis anterior (TA) muscle of mice and then allowed the animals to recover for various times. Muscle tissue was then harvested from the injected and contralateral untreated limbs. Consistent with our previous findings in mature mice, UFD2a-7/7a was the predominant isoform expressed in the untreated TA muscle (Fig. 1A). As previously reported, the injected TA muscle underwent transient degeneration, followed by regeneration, and return to the preinjected state [42]. In untreated muscle, UFD2a-7/7a was the predominant isoform expressed, consistent with our previous findings in mature mouse muscle (see Fig. 1A). Two days after CTX injection, expression of the mature muscle-specific UFD2a-7/7a isoform was undetectable, and that of the short isoform of UFD2a, which is expressed ubiquitously and in immature myoblasts, was markedly increased (Fig. 6A). Five days after CTX injection, UFD2a-7 and UFD2a-7/7a expression returned, and by 12 days, UFD2a-7 was almost undetectable, suggesting that UFD2a-7 is in fact a transient alternative splice form that may be important during the differentiation process. This switch in UFD2a isoform expression mirrored that of MHC, a well-known marker of mature muscle tissue. The transcription factor Myf5, which is expressed only in proliferating myoblasts, was not detected prior to injection, was induced at 2 to 5 days postinjection, and then returned to undetectable levels within 12 days, presumably upon completion of regeneration. These studies provide a compelling link between UFD2a isoform expression and the carefully regulated process of muscle regeneration.

### Sequential expression of UFD2a-7 and UFD2a-7/7a occurs during development

Since the molecular mechanisms that regulate adult myoblast differentiation are thought to be similar to those that regulate embryonic myogenesis, we analyzed UFD2a isoform expression during development in murine heart and skeletal muscle. As in differentiating myoblasts, the pattern of UFD2a alternative splice isoform expression appeared sequential, i.e., UFD2a-7 was expressed before UFD2a-7/7a. However, the ultimate switch to UFD2a-7/7a occurred much sooner in cardiomyocytes than in skeletal muscle. At embryonic day (E) 14, when primary skeletal muscle fibers have been established, Western blot analysis detected no UFD2a-7 or -7/7a protein in murine skeletal (Fig. 6B upper panels) or cardiac muscle tissue (Fig 6B lower panels). In skeletal muscle, UFD2a-7 was not detectable until E18, when secondary skeletal muscle myofibers were fusing. At P1, both UFD2a-7 and UFD2a-7/7a could be detected, as seen by the doublet in both tissue sets. By P7, UFD2a-7 was reduced and UFD2a-7/7a isoform dominated. Only a faint UFD2a-7 band was seen at E16, however, by E18 cardiomyocytes had already switched on UFD2a-7/7a expression, in contrast to skeletal muscle where UFD2a-7/7a expression did not become apparent until postnatal day (P1) .At all developmental time points tested, significantly more ubiquitous UFD2a isoform was present than was found in adult muscle tissue, which most likely indicates that myoblasts (in skeletal muscle) and other contaminating cells types (e.g., endothelial cells and fibroblasts) were actively dividing as the tissues continued to grow and develop. While the expression patterns discussed above were robust in western blots performed using samples from multiple mice, It was noted that there was some variability in the relative levels of each isoform detected at individual developmental time points. Therefore, a second set of skeletal muscle and heart tissue samples is shown in Figure S2.

To examine whether the sequential transition from Ufd2a-7 to UFD2a-7/7a during development is conserved, we examined the pattern of *ube4b* mRNA expression during early development in zebrafish by using RT-PCR. We specifically examined the mRNA expression pattern of *ube4b*, rather than protein levels, because neither rabbit polyclonal nor mouse monoclonal antibodies specifically recognized zebrafish Ube4b by Western blotting (as tested on *in vitro* transcribed and translated flag-tagged *Danio rario* UBE4B proteins, data not shown). Nested PCR performed on the purified products obtained from 10-, 12-, 24-, and 48-hpf (hours postfertilization) embryos and 7-dpf embryos using primers F1 and R1 followed by F2 and R2 (Fig. 3B) revealed that more of Ufd2a-7 was present at the earlier developmental stages (10 hpf –12 hpf) (Fig. 6C). These 2 nested PCR products were sequenced and blasted against the NCBI zebrafish *ube4b* genome. An identical match was found with 2 sequences within what had been previously considered intronic sequence in the zebrafish *UFD2a* ortholog. These results confirmed that the smaller amplicon was consistent with *Ube4b-7, and* the larger amplicon seen in increasing amounts from 12 hpf to 7 dpf represented the larger *Ube4b-7/7a* isoform. We have entered the two novel Ube4b isoforms in Genebank and the accession numbers: JF289275 (zebrafish UFD2a-7) and JF289276 (zebrafish UFD2a-7) have been assigned.

Interestingly, during the period between 10 hpf and 24 hpf, zebrafish somites and heart are forming, such that by 24 hpf a beating heart can be observed. These data provide evidence for the expression of both alternative splice forms of Ube4b during this period of zebrafish development in a sequential pattern like that seen in mammalian myogenesis.

### Muscle-specific UFD2a-7/7a fails to interact with VCP/p97

We hypothesized that the binding specificity of the muscle-specific isoform is altered from that of the ubiquitous UFD2a. UFD2a homologues in yeast, worms, and mice interact with the AAA-type ATPase VCP/p97 (cdc48 in yeast) [1], [23], [24], which functions in mitotic progression, muscle myofibril organization, and myoblast fusion [24]–[26]. Therefore, we sought to determine whether UFD2a-7/7a binds to VCP/p97 in a yeast 2-hybrid assay.

Mav203 yeast containing *Gal4*-regulated *HIS3* and *lacZ* genes were co-transformed with plasmids coding for a bait protein consisting of the *Gal4* DNA binding domain fused to either *UFD2a* or *UFD2a-7/7a* (pDEST32-UFD2a or pDEST32-UFD2a-7/7a) and a prey protein consisting of the *Gal4* activation domain fused to *VCP*/*p97* (pDEST22-VCP/p97). Yeast co-transformed with these plasmids were selected on double drop out medium and expression of the fusion proteins was confirmed by Western blot (Figure S3). Control yeast clones expressing the bait or prey proteins alone were co-transformed with empty pDEST32 or pDEST22.

**Figure 7:**
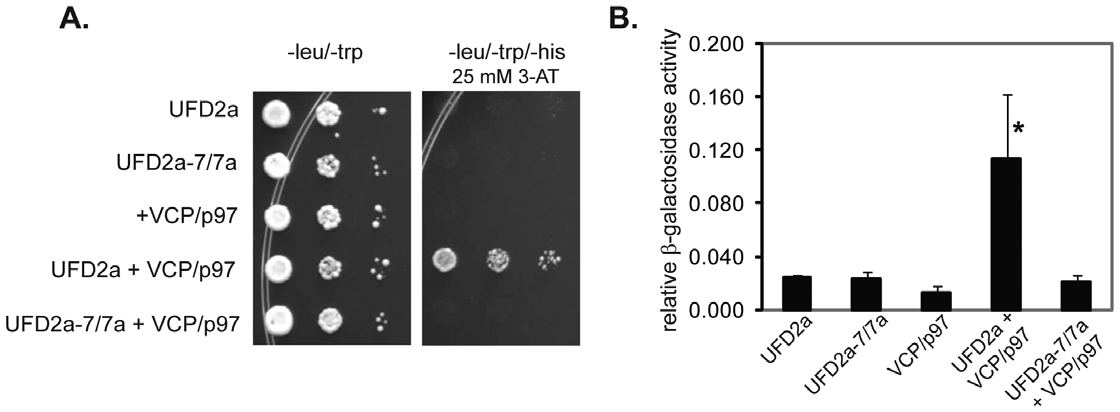
The muscle-specific UFD2a-7/7a isoform does not interact with VCP/p97. A). Yeast cells transformed with bait plasmids pDEST32-UFD2a, pDEST32-UFD2a-7/7a, or empty pDEST32 and prey plasmids pDEST22-VCP/p97 or empty pDEST22 were grown in liquid double dropout medium (-leu, -trp) to saturation and diluted to an OD_600_ of 1. Serial dilutions of these cell cultures were prepared in 96-well plates and transferred to triple-dropout agar plates (-leu, -trp, -his) supplemented with 25 mM 3-AT. B). Isolated colonies of yeast cells transformed (as in panel A) were grown to saturation, diluted to OD_600_ of 0.3, and grown to log phase (OD_600_ = 0.8-1.5). Cells were disrupted with glass beads in Z buffer; ONPG substrate and β-mercaptoethanol were added to the resulting supernatants. After incubation at 37°C, supernatants were transferred to 96-well plates and read at 420 nm. B). Relative β-galactosidase activity is calculated from 6 independent experiments; error bars indicate standard error. The average activity of UFD2a+VCP/p97 was significantly higher than that of the other transformants (*p = 0.02). The activity of UFD2a-7/7a+VCP/p97 did not differ from that of the negative controls.

Only yeast coexpressing UFD2a and VCP/p97 fusion proteins grew on medium lacking histidine in the presence of 3-aminotriazole, a histidine biosynthesis inhibitor, indicating an interaction between UFD2a and VCP/p97 fusion proteins. In contrast, neither yeast coexpressing UFD2a-7/7a and VCP/p97 fusion proteins nor yeast expressing bait or prey proteins alone grew in this medium (Fig. 7A). The lack of interaction between UFD2a-7/7a and VCP/p97 was confirmed by the absence of β -galactosidase activity in a liquid assay (Fig. 7B).

## Discussion

We describe here 2 muscle-specific alternative splice forms of the ubiquitin ligase, UFD2a. The smaller, UFD2a-7 contains the previously identified exon 7, while UFD2a-7/7a incorporates a novel exon 7a in addition to exon 7. Both of these alternatively spliced exons are located just downstream of the previously described MPAC regulatory domain and within the N-terminal extension of UFD2a, which is unique to vertebrate orthologs [28]. The GenBank database includes expressed sequence tag entries containing exon 7 spliced to exon 8 in human primary fibroblasts and thalamus tissue [43], and a full-length cDNA sequence of *UFD2a-7* cloned from a mouse fetal brain library. However, we did not detect UFD2a-7 protein in human or mouse fibroblast or brain tissues. Two recent genome-wide exon-junction and whole transcript microarrays, which examined the expression of alternative pre-mRNA splice forms across a total of 92 tissues and cell lines, both found that exon 7 was not present in UFD2a cDNA from human fetal brain tissue. In fact, consistent with the data presented here, exon 7 was exclusively expressed in adult human skeletal muscle, heart, tongue (therefore, predominantly in striated muscle cells) and to a lower extent peripheral blood leukocytes. Of the nine fetal tissues tested, only fetal heart expressed exon 7 (fetal skeletal muscle was not included). The tissue specificity of exon 7a was not examined since its presence in *UFD2a* had not yet been reported.

Our RT-PCR and Western blot data suggest that UFD2a-7 is uniquely expressed in a transient manner during myogenic development and myoblast differentiation. The timing of UFD2a-7 expression during differentiation and after injury appeared to correlate with that of the myogenic regulatory factors Myf5 and MyoD. Interestingly, ubiquitin-dependent degradation regulates most of these myogenic regulatory factors during myogenesis. The degradation of Myf5, in particular, is required for myoblast fusion [44] which occurs at the time of UFD2a-7 expression. In developing skeletal muscle, it coincided with the greatest expression of embryonic MHC (data not shown).

In cell culture models of differentiation and *in vivo* models of regeneration, UFD2a and UFD2a-7 protein persisted, most likely because differentiation was not complete. However, UFD2a-7 was not present in adult striated muscle tissue *in vivo*, and though small amounts of the ubiquitous UFD2a isoform were detected in tissue lysates, only UFD2a-7/7a was seen on Western blots of isolated EDL muscle fibers (data not shown). Therefore, UFD2a-7 expression is transient during muscle differentiation, a phenomenon which is mirrored during development, where UFD2a-7 was detected in both embryonic tissue from mice and zebrafish but disappeared by 7 days after birth.

The newly identified UFD2a-7/7a isoform was expressed with slightly delayed kinetics during differentiation *in vitro* and during regeneration and development *in vivo*, where its levels increased with time while UFD2a-7 decreased. Therefore, in both *in vitro* and *in vivo* models of myogenesis, the UFD2a alternatively spliced transcripts are expressed sequentially. The ubiquitous short isoform is likely expressed in dividing myoblasts due to its role in mitosis, while the transient expression of UFD2a-7 may be important for transitory events during differentiation such as myoblast fusion. Since expression of UFD2a-7/7a occurs late, it may have a unique function in terminal differentiation. Identification of the alternative splice form of Integrin α7β1, which appears late in differentiation [4], provides evidence that splicing can influence processes required for differentiation since α7β1 isoform specific protein-protein interactions regulate myotube formation and cell adherence in culture [45]. The fact that UFD2a-7/7a did not bind to VCP/p97 suggests that UFD2a alternative splice forms also have different binding specificities, strongly suggesting a unique function for UFD2a-7 and -7/7a in differentiated muscle cells.

Although several other muscle-specific ubiquitin E3 ligases have been identified, including F-box (MAFbx)/atrogin-1 and the muscle-specific RING finger (MURF) family, and their importance during development and muscle atrophy are well described [46], [47], [48], UFD2a is unique in that it represents a ubiquitously expressed ligase that has distinct alternative-splice forms in striated muscle. The central section of the exon 7a sequence is unusually rich in acidic amino acids. Although the length of this acidic sequence is variable among species, its presence is conserved. This finding suggests that the exon contains a unique binding domain that mediates interaction with a positively charged molecule. Interestingly, the N-terminal domain of UFD2a contains an RRRL motif, which mediates its interaction with VCP/p97. In fact, the yeast 2-hybrid data presented here showed that UFD2a-7/7a fails to bind VCP/p97, suggesting that the acidic stretch of exon 7a may disrupt UFD2a folding, affecting this interaction.

One striated muscle protein whose proteasome-dependent degradation depends on UFD2a and VCP/p97 is UNC-45b [19], [49]. *C. elegans* contains only one *unc-45* gene, but vertebrates express *UNC-45a* (also referred to as the General Cell (GC) form) in most tissues and *UNC45b* exclusively in striated muscle tissue [50]. UNC-45 proteins have myosin chaparonin activity which under certain conditions may involve forming a co-chaperone complex with HSP-90 [17], [51], [52], however recent studies in *C. elegans* suggest that UNC-45 function in muscle is inhibited by HSP-90 binding [53] UNC-45a appears to be essential to basic cellular processes such as cytokinesis, myoblast proliferation, and early fusion events, and UNC-45b is required for proper assembly of sarcomeric myosin filaments [17], [18], [50], [54], [55], [56]. Interestingly, the expression of UNC-45 isoforms mirrors those of UFD2a, where UNC-45a is expressed early during differentiation, and later its levels decrease while those of UNC-45b increase [50]. The degradation of human UNC-45b has been shown to be dependent upon human UFD2a and VCP/p97 [49], but the mechanism of UNC-45a degradation has not been studied. Potentially, UFD2a may not target UNC-45a for degradation, thereby allowing levels of this isoform to remain high during myoblast proliferation. However, at this time and during early stages of differentiation, UFD2a may ubiquitinate UNC-45b and target its degradation in a manner dependent on its interaction with VCP/p97. The importance of the regulation of UNC-45b levels is illustrated by the detrimental effects of UNC-45b overexpression. Increased Unc-45b in zebrafish results in disorganized myosin filaments [57], and overexpression of Unc-45 in worms leads to paralysis [18]. Later in differentiation, levels of UFD2a decrease concomitant with an increase in UFD2a-7 and -7/7a, the latter of which may not bind to VCP-p97, promoting UNC-45b degradation and subsequent accumulation of this vital chaperone. Moreover, UFD2a-7 may specifically target UNC-45a after the early myoblast fusion events have completed.

Like UFD2a, VCP/p97 has been implicated in the progression of cells through mitosis, a process that would be restricted to proliferating myoblasts, which express UFD2a-7. When VCP/p97 is bound to the UFD1-Npl4 cofactor complex, it extracts ubiquitinated Aurora B kinase from mitotic chromosomes, a process that is essential for decondensation, nuclear reformation, and spindle disassembly [58], [59]. If VCP/p97 were to interact with the UFD1-Nlp4 complex and UFD2a in a mutually exclusive fashion, UFD2a binding may inhibit premature extraction of Aurora B. The fact that both UFD2a and VCP/p97 are required for mitotic progression suggests that their interaction may be important only during myoblast proliferation. Later in differentiation, when myocytes have exited the cell cycle, the switching of UFD2a isoform expression to UFD2a-7/7a may promote complete differentiation by simply enabling VCP/p97 to bind other cofactors.

The differential binding of UFD2a and UFD2a-7/7a to VCP/p97 is also of interest given that mutations in this AAA-type ATPase are present in patients with inclusion body myopathy associated with Paget's disease of bone and frontotemporal dementia and cause complete disease pathology in transgenic mice [60], [61]. *In vitro* studies have suggested that VCP/p97 is crucial for homeostasis in mature muscle and during differentiation. Indeed, expression of disease-associated mutant VCP/p97 leads to an accumulation of ubiquitin-loaded autophagosomes and decreased myoblast fusion [62], [63].

In summary, the data reported here show that 2 alternatively spliced isoforms of the E3/4 ubiquitin ligase UFD2a, resulting from the inclusion of 2 exons highly conserved across vertebrate species, are specifically expressed in differentiating or mature striated muscle cells. Intron sequences immediately downstream of these muscle-specific exons contain recently described sequence motifs thought to convey muscle-specific alternative splicing. Given the sequential nature of isoform expression during zebrafish development and mammalian skeletal muscle differentiation and regeneration, we hypothesize that UFD2a-7 participates in key events of early myoblast differentiation, including the first cell-fusion event, and UFD2a-7/7a maintains the postmitotic state in mature muscle. We postulate that the differential binding capacity of these UFD2a isoforms to the ubiquitin-binding chaperone VCP/p97 is important to the regulation of UFD2a function during this process. These data are the first to suggest that UFD2a has divergent functions in striated muscle tissue and that elucidation of these functions during myocyte differentiation and physiology may provide further insight into the mechanisms of muscle regeneration and response to injury and disease.

## Materials and Methods

### RT-PCR

Total RNA was prepared from cells maintained in culture, murine tissues, and tissues extracted from male and female zebrafish (1–1.5 years old) or whole snap-frozen zebrafish embryos by using TRIzol (Invitrogen, Carlsbad, CA). RNA was converted to cDNA by using standard techniques. Human skeletal muscle total RNA was purchased (Stratagene, Santa Clara, CA). Aliquots of the cDNA were used as the template for PCR using a variety of primers specific for human *UFD2a*, murine *Ube4b*, or zebrafish *ube4b* sequences. *HPRT* served as a positive control. See table 1 for all primer sequences used.

**Table 1:** Primers used for PCR.

Organism	Fig.	Direction	Name/Gene	Applied Biosystems #	Sequence
Human	1D	Forward	*UFD2a*		GAGCATGGATATCGATGGTG
	1D	Reverse	*UFD2a*		GAGTGGTTCTCACCAGTTCC
	1D	Forward	*HPRT*		GCTGACCTGCTGGATTACAT
	1D	Reverse	*HPRT*		CCAGTTTCACTAATGACACAA
Mouse	5C		*UFD2a* exon 9	Mm00502528_m1	
	5C	Forward	*UFD2a* exon7a	Custom	TCCTCCCTGCGGATCTCT
	5C	Reverse			AGTTCGAGGAAGAGGAAGGAGAAA
	5C		*alpha actin*	Mm00808218_g1	
	5C		*MAFbx/Fbxo32*	Mm00499518_m1	
	5C		*HPRT*	Mm00446968_m1	
	5B 3^rd???^	Forward	*UFD2a* exon 6		CGAAGTCTAGACTGATTTTAAGGATCTGATTGGCC
	5B 3^rd^	Reverse	*UFD2a* exon 8		CATATCTCGAGCACTCGATGAGGTAGTTCG
	5B 1^st^ & 2^nd^	Forward	*UFD2a* exon 6		CTAAGTCTAGACTTCAGCACCAGCTCGTTGTCTAG
	5B 1^st^	Reverse	*UFD2a* exon 7-8 jct*		CATTACTCGAGCAGAGGCTCCCAAACTAGGAG
	5B 2^nd^	Reverse	*UFD2a* exon 7a-8 jct		CATATCTCGAGGCTCCCAAACTGGACCCAAAC
	5B 3rd	Forward	*UFD2a exon 19*		AGCTGATGCTGCGCATCCTGG
	5B 3rd	Reverse	*UFD2a exon 20*		GGAGATGGAACATGTCCACGG
Zebrafish	3C-D	Forward	*UBE4B* F1		GTAACCCATTCGCCAGCCTGAC
	3C-D	Reverse	*UBE4B* R1		ACAGCTGGTTGGCTGCACATC
	5C	Forward	*UBE4B* F2		CTCATGATGTCCACCCGTTC
	5C	Reverse	*UBE4B* R2		CAGTGGGAGCGAATGTTGCTC

*jct = junction.

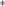
panels in figure 5B are 1–3 from top to bottom.

### Identification of exon 7 and 7a in zebrafish Ube4b

Total RNA was purified from zebrafish embryos at 10, 12, 24, and 48 hpf and 7 dpf. The cDNA was amplified using an oligodT primer (Invitrogen). Nested PCR was performed using 2 primer sets complementary to the end of exon 6 and beginning of exon 8 or 9 (according to NCBI accession AY029484). The inner primer pair contained *Hind*III restriction sites. PCR fragments were inserted into Blueskript SK(+) and sequenced (pBLUE-DR1 and pBLUE-DR2). Sequence data were blasted against the *D. rerio* genome database to confirm their localization within the *Ube4b* open reading frame on chromosome 23. See table 1 for all primer sequences.

### Quantitative PCR

Quantitative PCR reactions were performed using cDNA samples prepared as described above and TaqMan primer/probe sets. Prevalidated primer/probe sets (Applied Biosystems) were used for UFD2a (Mm00502528_m1), α-actin (Mm00808218_g1), MAFbx/Fbxo32 (Mm00499518_m1), and HPRT (Mm00446968_m1). The UFD2a primer/probe set targeted sequences within exon 9 and thus recognized all isoforms. A custom primer/probe set was used for UFD2a-7/7a. These sequences cross the exon 7/7a junction; thus, they recognize only transcripts containing both exons 7 and 7a. Transcript expression in each sample was normalized to the expression of *HPRT* by using the ΔΔCt method and was plotted relative to expression of that gene at time zero. See table 1 for all primer sequences.

### Western blotting

Lysates of cells in culture were prepared by scraping the cells into lysis buffer (10 mM Tris HCl, pH 7.6, 1% NP-40, 2 mM EDTA, 150 mM NaCl), supplementing the medium with 1 µg/mL pepstatin, leupeptin, antipain, and chymostatin and 1 mM phenylmethylsulfonylfluoride or 1×protease inhibitor cocktail (Pierce Chemicals, Rockford, IL). Tissue lysates were prepared by finely mincing tissue with a razor blade and then lysing it in a glass/Teflon homogenizer. Crude lysates were centrifuged for 10 min at 14,000 x *g* at 4°C, and the resulting supernatants were assayed for protein content. Cell and tissue lysates were treated with λ-protein phosphatase, according to the manufacturer's protocol, for 30 min at 30°C. Equal amounts of protein were electrophoresed on SDS-PAGE gels and then transferred to nitrocellulose membranes. UFD2a was detected using a polyclonal antibody that recognizes the N-terminal MPAC domain of UFD2a [28]. Other antibodies were from commercial sources: MHC (MF20, Developmental Studies Hybridoma Bank, University of Iowa, Iowa City, IA), α-sarcomeric actin (5C5, Sigma, St. Louis, MO), β-actin (AC-74, Sigma), myogenin (M-225, Santa Cruz Biotechnology, Santa Cruz, CA), Myf5 (C-20, Santa Cruz Biotechnology), vinculin and sarcomeric actin (Sigma), and β-tubulin (TUB 2.1, Sigma). Skeletal muscle tissue from E18 mouse embryos, P1 and P7 pups consisted of pooled gastrocnemius, soleus, and quadriceps muscles. That from E14 and E18 mice included some bone and skin. Tissue from 2 embryos or pups were collected at each time point and analyzed separately. Western blots shown in the supplementary information were performed using transformed yeast grown to log phase in –leu/-trp selective media. Cell pellets were washed in 1 ml of 20% Tricloroacetic acid (TCA), resuspended in 400 µl 20% TCA and 150 µl of acid washed glass beads and vortexed at 4°C. Supernatants were precipitated at 13 K and washed in 2% TCA. Protein pellets were resuspended in 200 µl of 1x SDS-PAGE sample buffer containing Tris base per original O.D_600_ of 2 and boiled. UFD2a and UFD2a-7/7a bait proteins fused to the Gal4 DNA binding domain (DB) were detected using a rabbit polyclonal antibody recognizing the Gal4 DB domain (Santa Cruz Biotechnology). VCP/p97 fused to the Gal4 activation domain (AD) was detected using a mouse monoclonal antibody recognizing the AD (Clontech). As an indicator of total protein loading the ATPase, Rpt5 was detected using a rabbit polyclonal antibody (Abcam).

### Human tissues

Deidentified frozen muscle biopsy samples were kindly provided by Dr. Andrea Corse (Johns Hopkins University, Baltimore, MD). De-identified frozen human heart and coronary artery samples were from explanted hearts of heart transplant recipients and were kindly provided by Dr. William Baldwin (Johns Hopkins University). Tissue lysates were prepared as described above. All human tissues used to make lysates were excess pathologic tissues and were obtained without identifiers under Title 45 CFR 46.101(b): “Research on Human Specimens", National Institutes of Health exemption #4 and therefore the need for consent was waived by the Johns Hopkins Institutional Review Board.

### Mouse tissue

Adult tissues were harvested from two male and one female C57Bl/6J mice, between 4 and 4.5 months of age. Lysates made from each mouse were analyzed on three separate western blots. This protocol (#38F-1) was approved by the Institutional Animal Care and Use Committee at Rhode Island College. Embryos and post-natal mice of various ages were harvested from timed-pregnant *C57Bl/6* female mice. Cardiac and skeletal muscle (gastrocnemius, soleus, and quadriceps) was dissected from three P7, P1, and E18 mice and embryos each, respectively. In order to isolate the same skeletal muscle groups from E14 and E16 embryos, entire hind limbs (-hind paws) were dissected from three embryos each, since skeletal muscle could not be distinguished from skin or bone in embryos at this stage of development. All mice were bred and maintained in accordance with the guidelines set forth by the National Institutes of Health Guide for the Care and Use of Laboratory Animals, published by the U.S. Public Health Service. This experimental protocol (protocol number 506) was approved by the Institutional Animal Care and Use Committee at St. Jude Children's Research Hospital.

### Zebrafish Embryos

All protocols for zebrafish husbandry and collection of embryos were approved by the Rhode Island College Institutional Animal Care and Use Committee under protocol number 34Sp-1.

### Muscle cell culture and differentiation

C2C12 mouse myoblasts were obtained from American Type Culture Collection (Manassas, VA) or as a generous gift from Dr. Robert Krauss (Mt. Sinai School of Medicine, New York, NY). C2C12 cells were maintained in DMEM high-glucose medium, supplemented with 10% fetal calf serum, 2 mM glutamine, 100 units/mL penicillin, and 100 µg/mL streptomycin. Differentiation was induced by culturing in differentiation medium in which the 10% fetal calf serum was replaced with 2% horse serum or 1×insulin/transferrin/selenium (10 mg/mL insulin, 5.5 mg/mL transferrin, and 6.7 ng/mL selenium). Primary human myoblasts (Cambrex, Walkersville, MD) were maintained in culture and differentiated according to the supplier's recommendations.

### Cardiotoxin-induced muscle atrophy model

Six-week-old C57BL/6 mice were anesthetized with isoflurane; the right legs were cleaned with alcohol; and the right TA muscles were injected with 0.1 mL CTX (10 mM) (Calbiochem, San Diego, CA) diluted in PBS. The contralateral uninjected TA muscles served as controls. On Days 1, 2, 3, 5, and 12 post–CTX injections, the mice were euthanized, and the bilateral TA muscles were removed. The muscles were frozen rapidly in dry ice–cooled isopentane and stored at –80°C. Samples were subsequently homogenized in lysis buffer and analyzed by Western blotting. These experiments were approved by the Johns Hopkins Animal Care and Use Committee, which follows the National Research Council and National Institutes of Health guidelines (protocol # MO06M41).

### Yeast 2-hybrid experiments

The cDNAs of *UFD2a* or *UFD2a-7/7a* and *VCP/p97* were cloned into plasmids pDEST-32 and pDEST-22, respectively, and transformed into the *S. cerevisiae* reporter strain MaV203 by using the ProQuest^TM^ Two-Hybrid System (Invitrogen). Double-transformants were selected on media lacking leucine and tryptophan; bait-only or prey-only controls were transformed with empty pDEST-32 or pDEST-22. Interactions were assessed by growth in the absence of histidine in the presence of 10, 25, 50, or 100 mM 3-aminotriazole histidine synthase antagonist, and by â-galactosidase assays. For the liquid β-galactosidase assays, cell cultures were diluted to an O.D._600_ of 0.3 in 3 mL media lacking leucine and tryptophan and grown to a final O.D._600_ of 0.8 to 1.5. After centrifugation, cells were resuspended in 300 µL Z buffer (60 mM NaH_2_PO_4_, 40 mM NaH_2_PO_4_, 10 mM KCl, 1 mM MgSO_4_, pH 7.0), disrupted by the addition of 150 mg autoclaved acid-washed glass beads (Sigma G8772), and vortexed for 30 minutes at 4°C. After brief centrifugation, supernatants were brought up to 1 mL total volume in Z buffer containing 25 mM β-mercaptoethanol and 2 mM ONPG (dissolved at 4 mg/mL Z buffer (N1127, Sigma) and incubated at 37°C. Reactions were stopped by the addition of 1 M Na_2_CO_3_, and centrifuged at 14,000 x g. Supernatants were transferred in triplicate into 96-well plates and their absorbance was read at 420. Relative β-galactosidase activity was calculated using the formula (1000×Abs)/(t_(min)_×O.D._600_). The statistical significance of the average activity of each cotransformant was analyzed using a 1-way analysis of variance (ANOVA) test of 6 independent experiments.

We thank Drs. Andrea Corse and William Baldwin (Johns Hopkins University) for providing human tissues for this study, David Hines (Johns Hopkins University) for technical support, and Dr. Robbert Creton (Brown University, Providence, RI) for assistance with the initial zebrafish studies.

## Supporting Information

Figure S1 **Ponceau S staining of proteins transferred to nitrocellulose used for the western blot in** **Figure 5A** **shows equal total protein loading (with the exception of the Day 0 sample).**
(PDF)

Figure S2 **The sequential expression of UFD2a isoforms during development in mice is robust across multiple samples.** A second set of mouse tissue lysates were made at various developmental time points (similar to Figure 6B). Skeletal muscle (upper panels; pooled gastrocnemius, soleus, and quadriceps muscles) or hearts (lower panels) from E14, E16, and E18 embryos and P1 and P7 pups were dissected from individual embryos or pups at each time point and analyzed by Western blotting for UFD2a. Equal total protein loading was assayed by western blotting for tubulin (bottom panels).
(PDF)

Figure S3 **Expression of bait and prey proteins in transformed MAV203 yeast cells can be detected by Western Blot.** Yeast cells were grown to log phase and precipitated proteins run on SD-PAGE gels. UFD2a bait proteins were detected using a polyclonal antibody recognizing the Gal4 DNA binding domain (DB) and the VCP prey protein was detected using a monoclonal anti-VCP antibody. Rpt5 was used as a loading control. Note that the anti-Gal4 DB antibody recognized high molecular weight proteins even in yeast not expressing a UFD2a bait protein (labeled non-specific).
(PDF)
