# Supplementary material for: A Novel Conserved Isoform of the Ubiquitin Ligase UFD2a/UBE4B Is Expressed Exclusively in Mature Striated Muscle Cells
Source: PLoS One. 2011 Dec 9;6(12):e28861. doi: 10.1371/journal.pone.0028861 (PMC3235170; doi:10.1371/journal.pone.0028861)
Supplement: Figure S1 — Ponceau S staining of proteins transferred to nitrocellulose used for the western blot in Figure 5A shows equal total protein loading (with the exception of the Day 0 sample). (PDF) [file pone.0028861.s001.pdf]

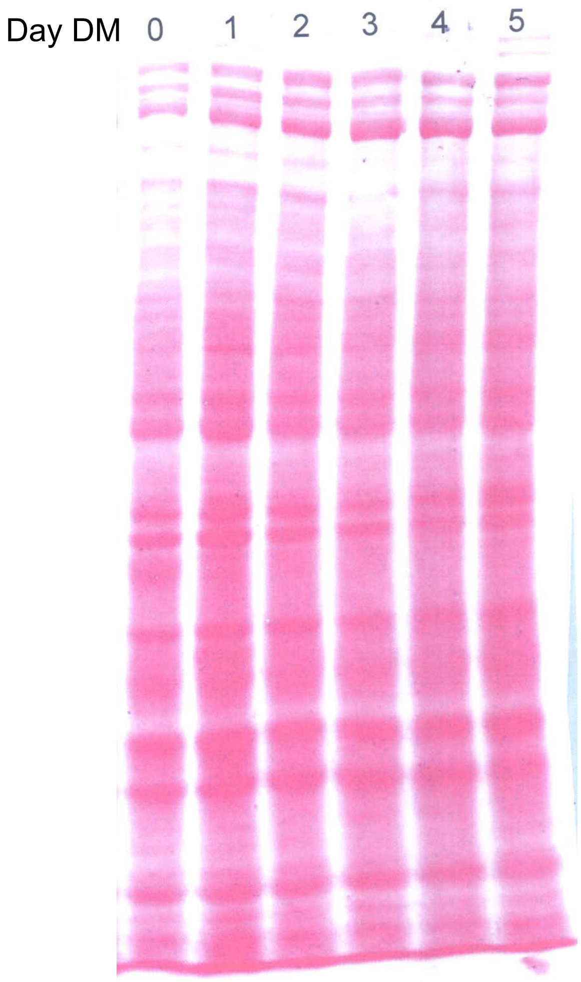

Supporting Information Figure S1: Ponceau S staining of proteins transferred to nitocellulose used for the western blot in Figure 5A shows equal total protein loading (with the exception of the Day 0 sample).
