# Supplementary material for: A Novel Conserved Isoform of the Ubiquitin Ligase UFD2a/UBE4B Is Expressed Exclusively in Mature Striated Muscle Cells
Source: PLoS One. 2011 Dec 9;6(12):e28861. doi: 10.1371/journal.pone.0028861 (PMC3235170; doi:10.1371/journal.pone.0028861)
Supplement: Figure S2 — The sequential expression of UFD2a isoforms during development in mice is robust across multiple samples. A second set of mouse tissue lysates were made at various developmental time points (similar to Figure 6B). Skeletal muscle (upper panels; pooled gastrocnemius, soleus, and quadriceps muscles) or hearts (lower panels) from E14, E16, and E18 embryos and P1 and P7 pups were dissected from individual embryos or pups at each time point and analyzed by Western blotting for UFD2a. Equal total protein loading was assayed by western blotting for tubulin (bottom panels). (PDF) [file pone.0028861.s002.pdf]

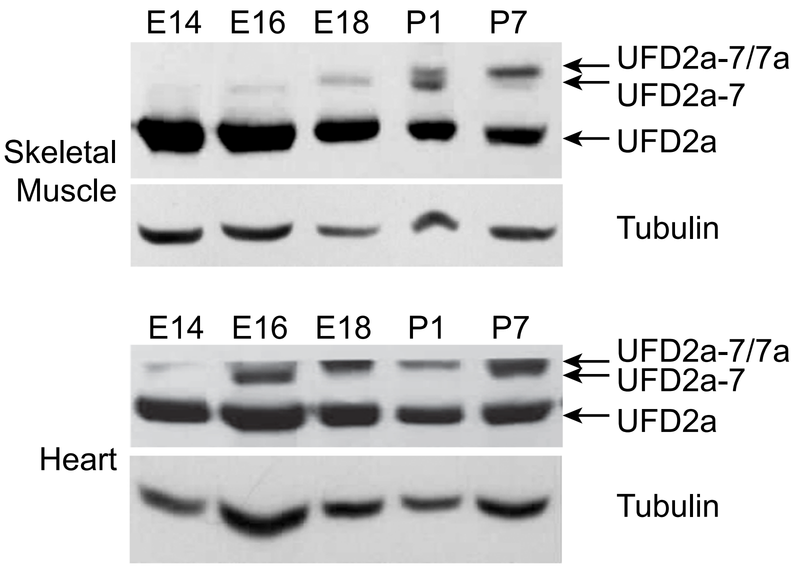

Figure S2. The sequential expression of UFD2a isoforms during development in mice is robust across multiple samples. A second set of mouse tissue lysates were made at various developmental time points (similar to Figure 6B). Skeletal muscle (upper panels; pooled gastrocnemius, soleus, and quadriceps muscles) or hearts (lower panels) from E14, E16, and E18 embryos and P1 and P7 pups were dissected from individual embryos or pups at each time point and analyzed by Western blotting for UFD2a. Equal total protein loading was assayed by western bloting for tubulin (bottom panals).
