# Supplementary material for: A Novel Conserved Isoform of the Ubiquitin Ligase UFD2a/UBE4B Is Expressed Exclusively in Mature Striated Muscle Cells
Source: PLoS One. 2011 Dec 9;6(12):e28861. doi: 10.1371/journal.pone.0028861 (PMC3235170; doi:10.1371/journal.pone.0028861)
Supplement: Figure S3 — Expression of bait and prey proteins in transformed MAV203 yeast cells can be detected by Western Blot. Yeast cells were grown to log phase and precipitated proteins run on SD-PAGE gels. UFD2a bait proteins were detected using a polyclonal antibody recognizing the Gal4 DNA binding domain (DB) and the VCP prey protein was detected using a monoclonal anti-VCP antibody. Rpt5 was used as a loading control. Note that the anti-Gal4 DB antibody recognized high molecular weight proteins even in yeast not expressing a UFD2a bait protein (labeled non-specific). (PDF) [file pone.0028861.s003.pdf]

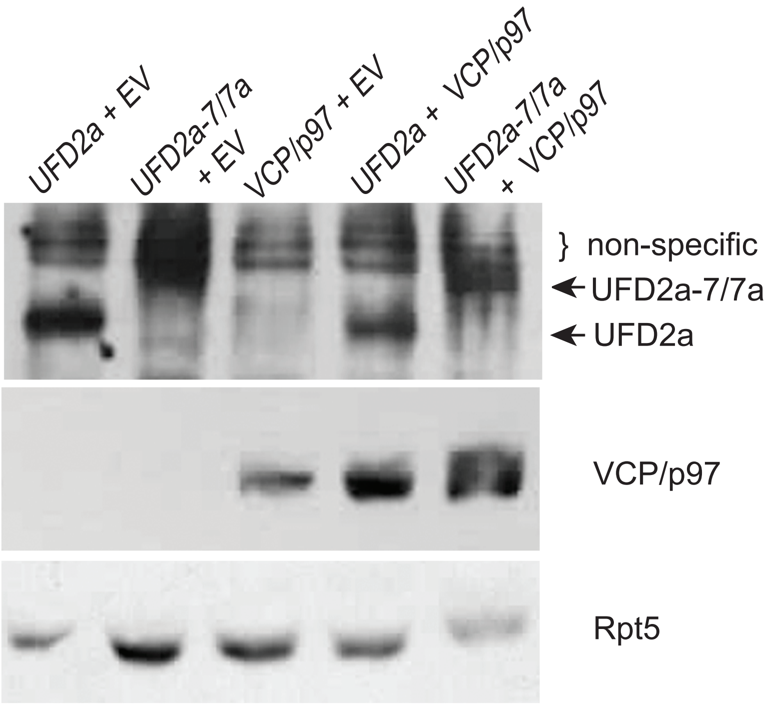

Supplementary Figure S3: Expression of bait and prey proteins in transformed MAV203 yeast cells can be detected by Western Blot. Yeast cells were grown to log phase and precipitated proteins run on SD-PAGE gels. UFD2a bait proteins were detected using a polyclonal antibody recognizing the Gal4 DNA binding domain (DB) and the VCP prey protein was detected using a monoclonal anti-VCP antibody. Rpt5 was used as a loading control. Note that the anti-Gal4 DB antibody recognized high molecular weight proteins even in yeast not expressing a UFD2a bait protein (labeled non-specific).
